# Anti-arthritic, immunomodulatory, and inflammatory regulation by the benzimidazole derivative BMZ-AD: Insights from an FCA-induced rat model

**DOI:** 10.1515/biol-2025-1083

**Published:** 2025-06-17

**Authors:** Haseeb Ahmad, Irfan Anjum, Halima Usman, Aisha Mobashar, Arham Shabbir, Yousef A. Bin Jardan, Amira Metouekel, Musaab Dauelbait, Mohammed Bourhia

**Affiliations:** Faculty of Pharmacy, The University of Lahore, Lahore, Pakistan; Department of Basic Medical Sciences, Shifa College of Pharmaceutical Sciences, Shifa Tameer-e-Millat University, Islamabad, Pakistan; Faculty of Health Sciences, Equator University of Science and Technology, Maska, Uganda; Department of Pharmacology, Institute of Pharmacy, Faculty of Pharmaceutical and Allied Health Sciences, Lahore College for Women University, Jail Road, Lahore, Pakistan; Department of Pharmaceutics, College of Pharmacy, King Saud University, P.O. Box 11451, Riyadh, Saudi Arabia; University of Technology of Compiègne, EA 4297 TIMR, 60205, Compiègne Cedex, France; College of Public and Environmental Health, University of Bahr el Ghazal, Freedowm Street, Wau, 91113, South Sudan; Laboratory of Biotechnology and Natural Resources Valorization, Faculty of Sciences, Ibn Zohr University, 80060, Agadir, Morocco

**Keywords:** rheumatoid arthritis, benzimidazole derivative, inflammation, immunomodulatory, pro-inflammatory cytokines

## Abstract

Rheumatoid arthritis (RA) is an autoimmune disease causing joint inflammation, deformity, cartilage deterioration, and pain. Benzimidazole derivatives exhibit various pharmacological properties. This study evaluated the antiarthritic, immunomodulatory, and anti-inflammatory potential of a benzimidazole derivative 2-(2-(benzylthio)-1*H*-benzo[d]imidazol-1-yl)-*N*′-(4-nitrobenzylidiene) acetohydrazide (BMZ-AD) in a Freund’s complete adjuvant (FCA)-induced arthritic rat model. FCA was administered on day 0, and treatment with BMZ-AD (25 mg/kg, 50 mg/kg, and 75 mg/kg) and piroxicam (10 mg/kg) began on day 7 and continued up to 28 days. Rats were sacrificed on day 28. Arthritis was assessed using an arthritic scoring index, and paw edema was measured with a digital water plethysmometer. Biochemical and hematological parameters were analyzed, reverse transcription polymerase chain reaction measured the mRNA expression of tumor necrosis factor alpha (TNF-α) and interleukin-6 (IL-6), and molecular docking evaluated BMZ-AD interactions with these proteins. Enzyme-linked immunosorbent assay determined prostaglandin E2 levels. BMZ-AD treatment reduced inflammation, pannus formation, and pro-inflammatory cytokines (TNF-α and IL-6) and decreased PGE2 levels, comparable to piroxicam. Blood profiles improved with significant reductions in white blood cells and platelets in treatment groups. BMZ-AD demonstrated antiarthritic, anti-inflammatory, and immunomodulatory properties, suggesting that it could be a potential drug for RA treatment with fewer side effects.

## 1 Introduction

Rheumatoid arthritis (RA) is a chronic autoimmune disorder characterized by joint cartilage erosions, bone remodeling, and synovium inflammation. Around 1% of the world population has been afflicted by the chronic inflammatory joint disease RA and the resultant disability [1]. Environmental and genetic factors contribute to the disease pathology. It is gender specific and four times more prevalent in females than in males. The cardinal features of RA include joint stiffness, severe pain, and functional disability which worsen in early morning [2].

RA develops with the inflammation of the synovial membrane, known as synovitis, which leads to joint swelling and pain during movement. A high number of immune cells move to synovial fluids in response to joint inflammation. Later on, the inflammation extends to the synovial tissue, joint cavity spaces, and cartilages. Pannus formation in synovium culminates in loss of joint cartilage. The condition aggravates further with fibrous tissue formation and fusion of bones [3].

Macrophages, T and B lymphocytes, fibroblasts, and a myriad of pro-inflammatory cytokines, notably interleukin-6 (IL-6) and TNF-α (tumor necrosis factor alpha), in conjunction with prostaglandin E2 (PGE2), are pivotal actors in the intricate pathogenesis of RA, contributing to the hyperplasia of articular cartilage. As a primary inducer of the production of acute-phase proteins, IL-6 orchestrates acute inflammatory responses, amplifying the cascade of immune-mediated processes characteristic of RA [4].

The five classes of drugs currently used in treatment include nonsteroidal anti-inflammatory drugs (NSAIDs), glucocorticoids, disease-modifying anti-rheumatoid drugs, and anti-cytokine therapies [5]. However, these therapies are associated with several undesirable effects such as irritation of stomach lining, gastrointestinal bleeding, weight gain, hypertension, increased blood sugar level, and loss of bone density. Since inflammation is the main culprit in the pathology of RA, agents having anti-inflammatory and antioxidant potential could be beneficial in RA therapy. A plethora of studies corroborate the anti-inflammatory potential of benzimidazole derivatives in various diseases [6,7,8,9].

The benzimidazole nucleus, often hailed as the “Master Key” in the realm of medicinal compounds, holds a pivotal and multifaceted role in drug development. By altering the benzimidazole structure at various positions, researchers can notably enhance the physicochemical, metabolic, and pharmacokinetic properties of the synthesized compounds. A host of benzimidazole-containing drugs have garnered FDA approval, including omeprazole, a proton pump inhibitor, and albendazole, an antifungal agent [10].

Benzimidazole is remarkable for its broad spectrum of biological activities. It showcases antifungal [11], anthelmintic [12], and anti-asthmatic properties [13]. Moreover, it acts as an anti-diabetic [14], antihypertensive [15], and antiparasitic agent [16]. Its antihistaminic [17] and gastroprotective capabilities [18] are also noteworthy, alongside its antibacterial [19], anticoagulant [20], and anti-obesity effects [21]. Furthermore, benzimidazole exhibits significant antioxidant [22], antitumor [23], and anti-inflammatory activities [24].

The versatility and breadth of action underscore the importance of the benzimidazole nucleus in medicinal chemistry, making it a cornerstone in the development of new therapeutic agents. On the basis of its significant anti-inflammatory potential, this study was designed to investigate its immunomodulatory role in arthritis. In this research study, we evaluated the mechanistic effects of a benzimidazole derivative [2-(2-(benzylthio)-1*H*-benzo[d]imidazol-1-yl)-*N*′-(4-nitrobenzylidiene) acetohydrazide (BMZ-AD) in a Freund’s complete adjuvant (FCA)-induced arthritic rat model. The results revealed that the benzimidazole derivative attenuated joint inflammation, bone erosion, and cartilage erosion. Moreover, its immunomodulatory role was attributed to the downregulation of pro-inflammatory markers such as TNF-α, IL-6, and PGE2 levels. Thus, this study provides valuable insights into discovering a benzimidazole derivative as a novel therapeutic drug for this challenging autoimmune disorder.

## 2 Materials and methods

### 2.1 Animals

Thirty-six healthy albino male rats weighing 250–350 g were procured from the Central Animal House of University of Lahore and divided into six equal groups, each containing six rats. Animals were kept in propylene cages having a stainless steel cover lid, under the conditions of ambience temperature of 22–25°C and a 12-h light/dark cycle, with free access to food and clean water. The rats were acclimatized to the laboratory conditions for 1 day before the experiment. The study protocol was approved by the Institutional Research Ethics Committee (IREC) at the University of Lahore prior to the commencement of the experiments, under Trial Registry No: IREC-PHM-21-46 [23].


**Ethical approval:** The research related to animal use complied with all the relevant national regulations and institutional policies for the care and use of animals and was approved by the Institutional Research Ethics Committee (IREC) at the Faculty of Pharmacy, the University of Lahore, Pakistan (No: IREC-PHM-2021-46).

### 2.2 Induction and assessment of arthritic progression

Rats were divided into six groups each containing six rats. **Group I** – vehicle control administered with 1% Tween 80 and 2% DMSO in distilled water. **Group II** – disease group administered with FCA (0.15 ml), **Group III** – reference group that received piroxicam (10 mg/kg b.w., p.o.), **Group-IV** – treatment group that received the test drug (BMZ-AD) 25 mg/kg b.w., p.o., **Group-V** – treatment group that received BMZ-AD 50 mg/kg b.w., p.o., and **Group-VI** – treatment group that received BMZ-AD 75 mg/kg b.w., p.o. At day 0, FCA was injected into the sub-plantar region of right hind paws of rats of all groups except control. From day 7 to day 28, BMZ-AD was administered to all test drug treatment groups (**Group-IV**, **Group-V**, and **Group-VI)**, and piroxicam was administered to **Group III** [25,26]. Inflammation, redness, and erythema were noted on days 7, 14, 21, and 28. Macroscopic observations of paw inflammation, redness, and swelling were used to determine the arthritic score. Redness, erythema, and inflammation of the paws were graded on a scale of 0, 1, 2, 3, and 4, which indicated normal, minimal, mild, moderate, and severe, respectively [27]. A digital water displacement plethysmometer was used to measure the paw edema of all groups at days 0, 8, 15, 22, and 28 [28].

### 2.3 Investigations of histopathology

Albino rats were sacrificed on day 28 and ankle joints were dissected. They were cut into ihalf lengthwise and preserved in 10% formalin and were subjected to decalcification by methanoic acid. After fixing in paraffin blocks, tissue slides of bisected paws were prepared [29]. The prepared slides were deparaffinized using xylene, rehydrated using gradient alcohol concentration (95–70%), and finally with distilled water. These slides were then stained with the hematoxylin and eosin (H&E) dyes and blindly evaluated by a histo-pathologist.

### 2.4 Analysis of mRNA expression levels of TNF-α and IL-6

The mRNA expression levels of cytokines involved in the development of RA were evaluated using the conventional polymerase chain reaction methodology. Initially, blood samples were collected through intracardiac puncture after sacrificing the rats in order to extract RNA, using TRIzol reagent in accordance with the established technique. For homogenization, 200 µl blood was mixed with 600 µl TRIzol to extract mRNA. Quantification of the isolated RNA samples was performed using a NanoDrop spectrophotometer. The complementary DNA was synthesized in accordance with the methodology provided by the kit manufacturer (Thermo Scientific, America). GAPDH (glyceraldehyde-3-phosphate dehydrogenase) was used as the reference. A sequence of primers for genes such as IL-6 and TNF-α was selected from previously published studies [30,31].

**Table:** 

Genes	Forward primer	Reverse primer
IL-6	5′-CCCACCAAGAACGATAGTCA-3′	5′-CTCCGACTTGTGAAGTGGTA-3′
TNF-α	5′-AGTCCGGGCAGGTCTACTTT-3′	5′-GGAAATTCTGAGCCCGGAGT-3′

### 2.5 Assessment of PGE2 through ELISA

PGE2 levels were detected from serum using Elabscience’s enzyme-linked immunosorbent assay (ELISA) kit (E-EL-003 96T). Absorbance was measured using an ELISA reader (BioTek Instruments, Inc., ELx808IU, USA) at 450 nm wavelength [32].

### 2.6 Evaluation of hematological parameters

A cardiac puncture was used to collect blood samples, which were then placed in a Vacutainer (Lab Vac) with EDTA. Hematological parameters, such as hemoglobin (Hb), red blood cells (RBCs), platelets (PLT), and white blood cells (WBCs), were analyzed with the help of an automated hematology analyzer (Sysmex XT-1800i) [33].

### 2.7 Evaluation of biochemical markers

Serum was separated from blood and was used for evaluation of biochemical parameters. Different biochemical parameters such as urea, creatinine, aspartate aminotransferase (AST), alanine transaminase (ALT), and alkaline phosphatase (ALP) were analyzed using an automated chemistry analyzer (HumaLyzer 3500) by following kit manufacturer’s protocols [30,34].

### 2.8 *In silico* studies

#### 2.8.1 Preparation of target proteins

**Figure 1:**
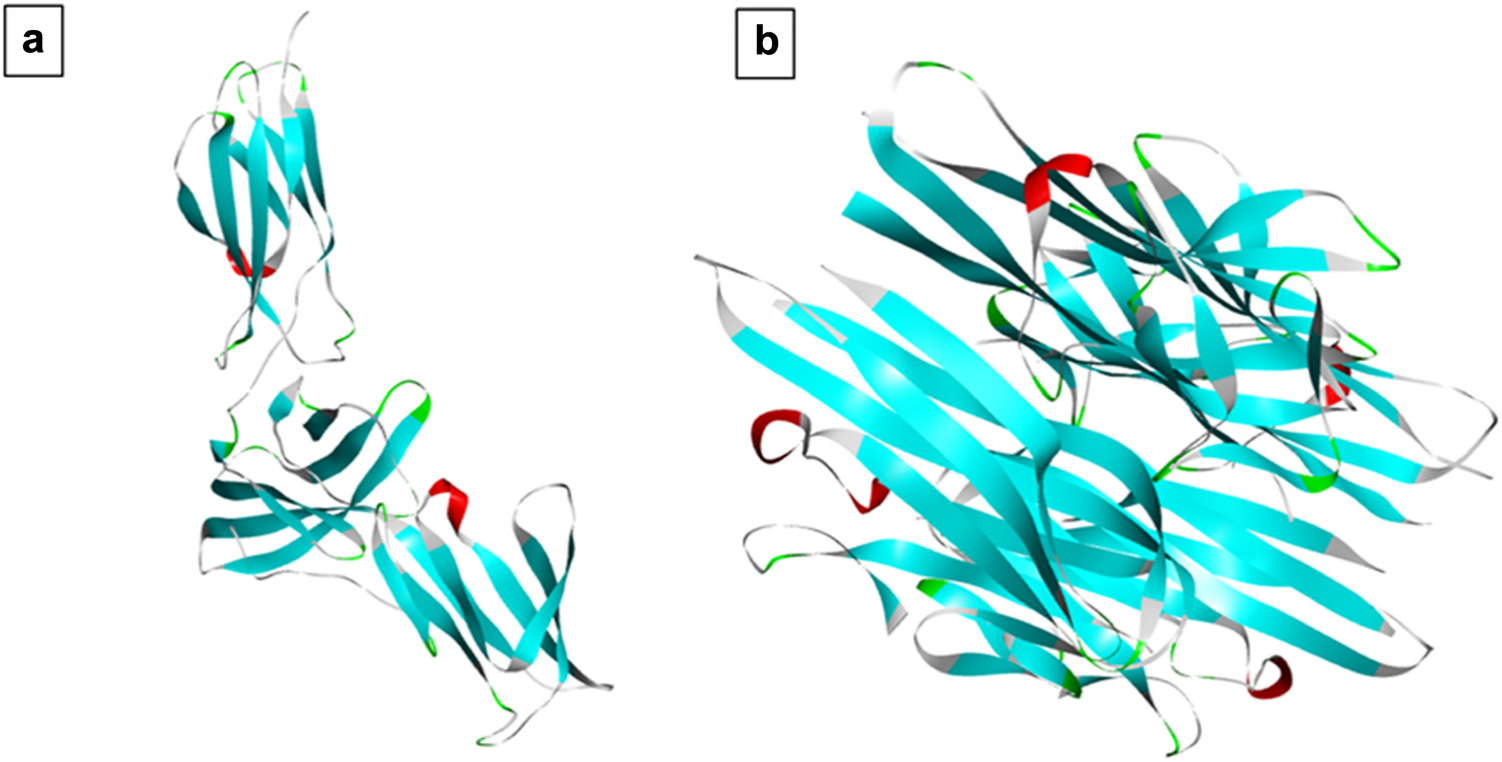
Three-dimensional (3D) X-ray crystal structures of (a) IL-6 (PDB ID: 1N26) and (b) TNF-α (PDB ID: 2AZ5).

The 3D crystal structures of target protein molecules were retrieved from protein data bank, PDB ID: 1N26 (IL-6), having a resolution of 2.40 Å, and PDB ID: 2AZ5 (TNF-α) having a resolution of 2.10 Å (Figure 1). The active binding sites of IL-6 and TNF-α were identified through literature review [35,36]. Processing of target protein was done using Discovery Studio Visualizer. Any co-crystallized ligands were removed and polar hydrogen atoms were added and saved in pdb format [37].

#### 2.8.2 Preparation of ligands

**Figure 2:**
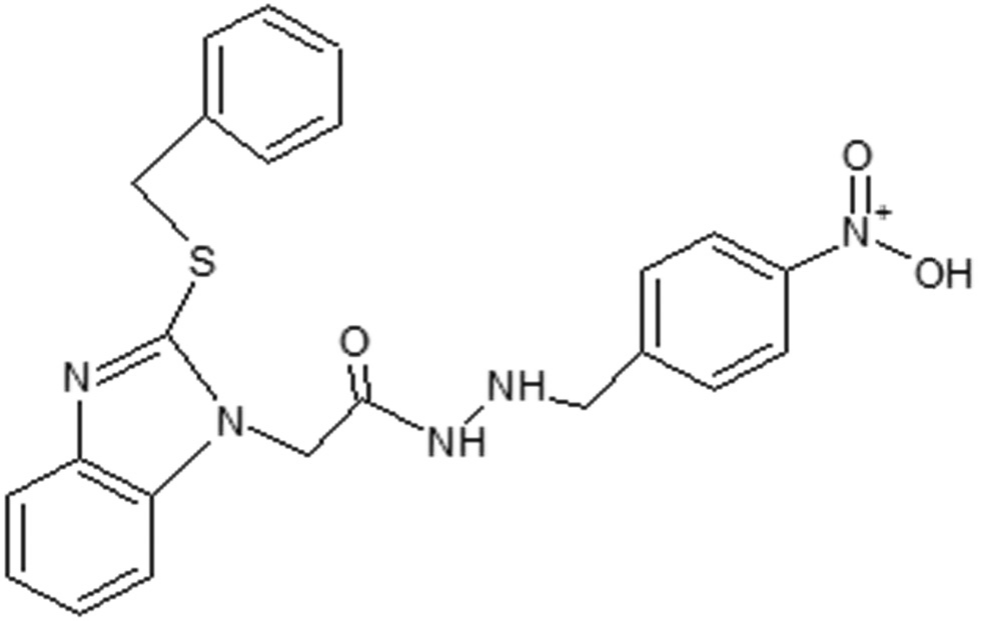
2D structure of BMZ-AD.

The 3D structure of piroxicam was downloaded from the PubChem database in sdf format, while the 2D structure of BMZ-AD was drawn in ChemSketch (Figure 2) and converted to pdb format using Discovery Studio Visualizer. Energy optimization and conversion to pdbqt format were done using Open Babel in PyRx software [38].

#### 2.8.3 Molecular docking

Molecular docking studies were executed using AutoDock Vina 4.2 suite of PyRx software. The prepared proteins and ligands were used as inputs in PyRx. The number of runs was set to 100 for each docking. The grid boxes were created around the amino acids of IL-6 (Glu144, Asn226, Asn110, and Gln158) and TNF-α (Leu57, Tyr59, Ser60, Gln61, Tyr119, Leu120, Gly121, Gly122, and Tyr151). The output docking scores were defined as the binding affinity (kcal/mol). To visualize the interaction of target amino acid residues with selected ligands, Discovery Studio Visualizer was utilized. The results obtained were expressed as the number of conventional hydrogen bonds with amino acid residues and binding affinity as kcal/mol [8,39].

### 2.9 Statistical analysis

For data interpretation, GraphPad Prism (v 6.0) was used. Values were presented as mean and standard error of mean (SEM). The results were analyzed by one-way analysis of variance (ANOVA), followed by Tukey’s *post-hoc* comparison test. *P* < 0.05 was set as the significance level.

## 3 Results

### 3.1 Effect of BMZ-AD on arthritic score

**Figure 3:**
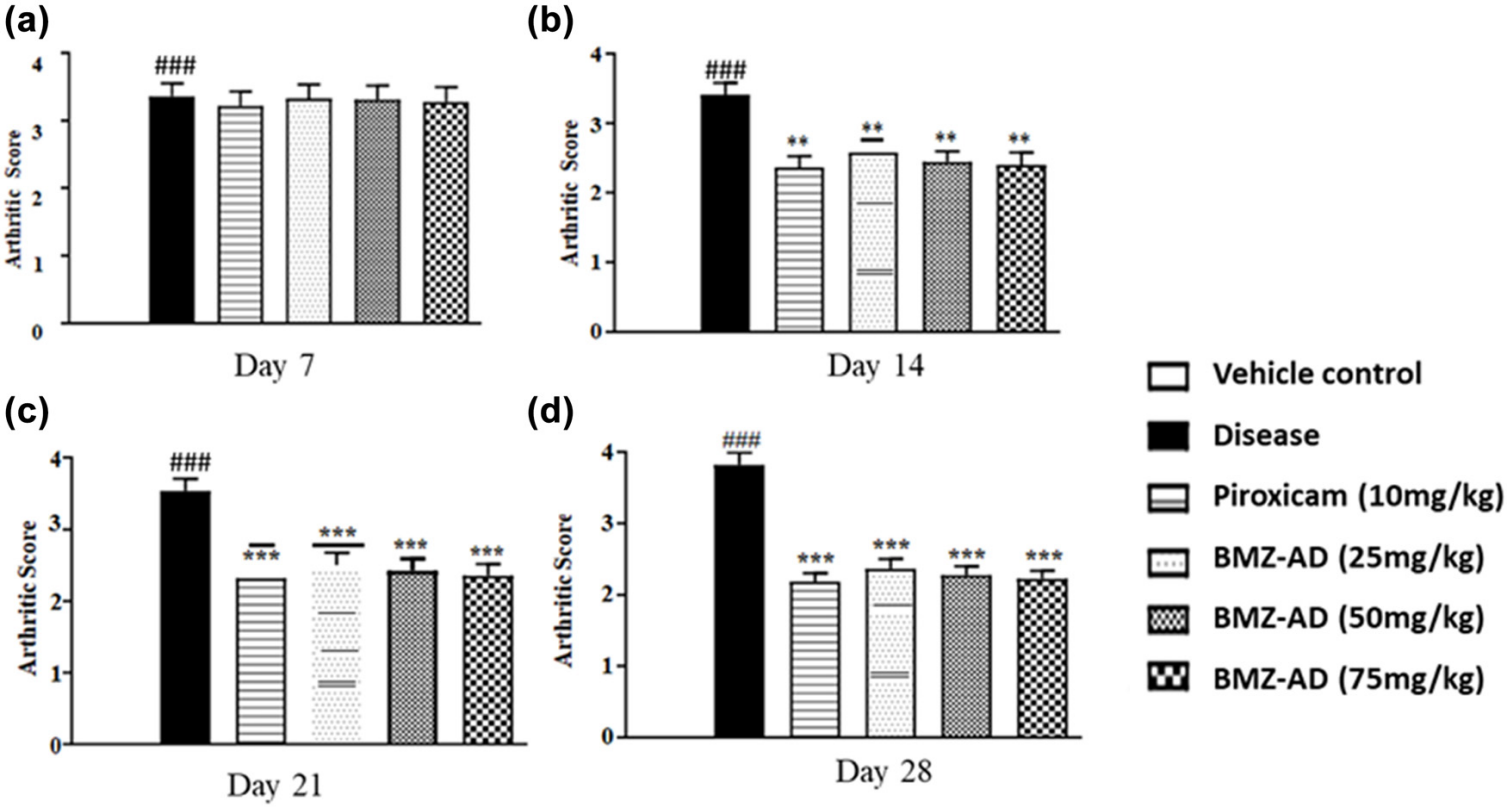
Effect of treatment on arthritic score with piroxicam 10 mg/kg and BMZ-AD (25, 50, and 75 mg/kg) on (a) 7th day, (b) 14th day, (c) 21st day, and (d) 28th day. The data are expressed as mean ± SEM, *** and ### indicate *p* < 0.001 and ** indicates *p* < 0.01. ###*p* < 0.001 vs vehicle control, while ***p < 0.001 and **p < 0.01 vs disease group.

On day 7, no significant effect on arthritic score was observed between piroxicam (3.221 ± 0.087), BMZ-AD 25 mg/kg (3.326 ± 0.084), BMZ-AD 50 mg/kg (3.306 ± 0.085), and BMZ-AD 75 mg/kg (3.281 ± 0.089) treatment groups and the disease group (Figure 3) (Table 1). After 28 days, substantial inhibition in arthritic score was observed with piroxicam (2.191 ± 0.048), BMZ-AD 25 mg/kg (2.370 ± 0.055), BMZ-AD 50 mg/kg (2.281 ± 0.049), and BMZ-AD 75 mg/kg (2.224 ± 0.044) treatment groups as compared to the disease group (3.822 ± 0.071).

**Table 1:** BMZ-AD significantly decreased arthritic development

Days	Disease	Piroxicam (10 mg/kg)	BMZ-AD (25 mg/kg)	BMZ-AD (50 mg/kg)	BMZ-AD (75 mg/kg)
Mean ± SEM (ml)					
Day 7	3.35 ± 0.079	3.22 ± 0.087	3.32 ± 0.084	3.30 ± 0.085	3.28 ± 0.089
Day 14	3.41 ± 0.069	2.36 ± 0.067***	2.58 ± 0.054***	2.44 ± 0.064***	2.40 ± 0.073***
Day 21	3.53 ± 0.067	2.32 ± 0.062***	2.50 ± 0.071***	2.43 ± 0.067***	2.36 ± 0.064***
Day 28	3.82 ± 0.071	2.19 ± 0.048***	2.37 ± 0.055***	2.28 ± 0.049***	2.22 ± 0.044***

In contrast to the disease group, BMZ-AD showed a significant difference of ***p < 0.001.

### 3.2 Effect of BMZ-AD on histopathological evaluation

#### 3.2.1 Inflammation was significantly reduced by BMZ-AD

**Figure 4:**
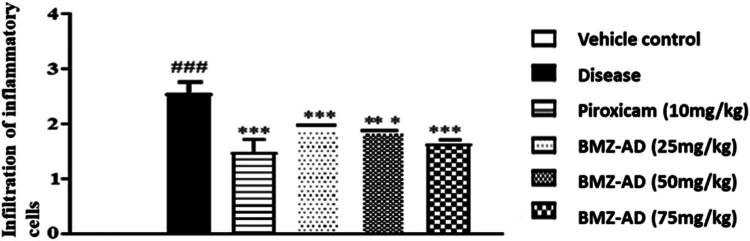
Infiltration of inflammatory cells with piroxicam 10 mg/kg and BMZ-AD (25, 50, and 75 mg/kg). The data are expressed as mean ± SEM, ***P < 0.001 vs disease group. Vehicle group data are shown as 0.

In contrast to the disease group (2.567 ± 0.080), there was a substantial reduction in inflammation in piroxicam 10 mg/kg (1.489 ± 0.093; *P* < 0.001), BMZ-AD 25 mg/kg (1.930 ± 0.028; *P* < 0.001), BMZ-AD 50 mg/kg (1.836 ± 0.029; *P* < 0.001), and BMZ-AD 75 mg/kg (1.634 ± 0.030; *P* < 0.001) treatment groups (Figure 4 and Table 2).

**Table 2:** BMZ-AD effect on histopathological parameters

Parameters	Disease	Piroxicam (10 mg/kg)	BMZ-AD (25 mg/kg)	BMZ-AD (50 mg/kg)	BMZ-AD (75 mg/kg)
Mean ± SEM					
Infiltration of inflammatory cells	2.56 ± 0.080	1.48 ± 0.093***	1.93 ± 0.028***	1.83 ± 0.029***	1.63 ± 0.030***
Pannus formation	3.61 ± 0.068	2.37 ± 0.094***	2.63 ± 0.02***	2.61 ± 0.024***	2.57 ± 0.011***
Bone erosion	2.65 ± 0.070	2.16 ± 0.063***	2.17 ± 0.066***	2.16 ± 0. 066***	2.16 ± 0.058***

BMZ-AD were shown to have a ***P < 0.001 difference in contrast to the disease group.

#### 3.2.2 Effect of BMZ-AD on pannus formation

**Figure 5:**
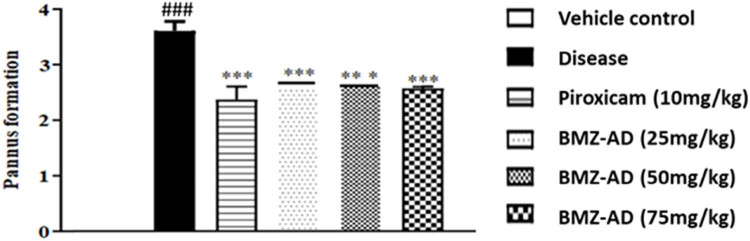
Effect of BMZ-AD on pannus formation after treatment with piroxicam 10 mg/kg and BMZ-AD (25, 50, and 75 mg/kg) on day 28. ###*P* < 0.001 vs vehicle group and ***P < 0.001 vs disease group.

Pannus formation was significantly augmented in the disease group as compared to the vehicle group. In contrast to the disease group (3.611 ± 0.068), piroxicam 10 mg/kg (2.374 ± 0.094), BMZ-AD 25 mg/kg (2.584 ± 0.083), BMZ-AD 50 mg/kg (2.665 ± 0.105), and BMZ-AD 75 mg/kg (2.581 ± 0.083) treatment groups showed a reduction (*P* < 0.001) in pannus development (Figure 5 and Table 2).

#### 3.2.3 BMZ-AD significantly suppressed bone erosion

**Figure 6:**
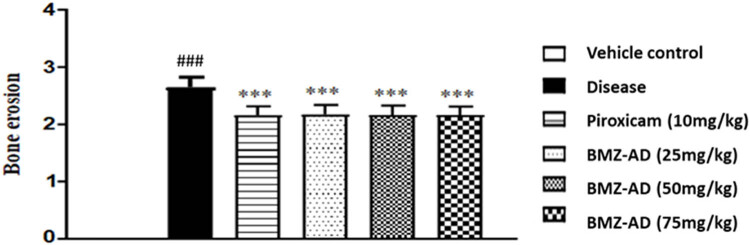
BMZ-AD reduced bone erosion after treatment with piroxicam 10 mg/kg and BMZ-AD (25, 50, and 75 mg/kg) on day 28. The data are expressed as mean ± SEM, ###*P* < 0.001 vs vehicle group and ***P < 0.001 vs disease group. Normal group data are shown as zero.

In contrast to the disease group, the piroxicam 10 mg/kg (2.164 ± 0.063; *P* < 0.001), BMZ-AD 25 mg/kg (2.177 ± 0.066; *P* < 0.001), BMZ-AD 50 mg/kg (2.166 ± 0.066; *P* < 0.001), and BMZ-AD 75 mg/kg (2.169 ± 0.058; *P* < 0.001) treatment groups showed a significant decrease in bone erosion (Figure 6) (Table 2).

### 3.3 Effect of treatment on histopathology

**Figure 7:**
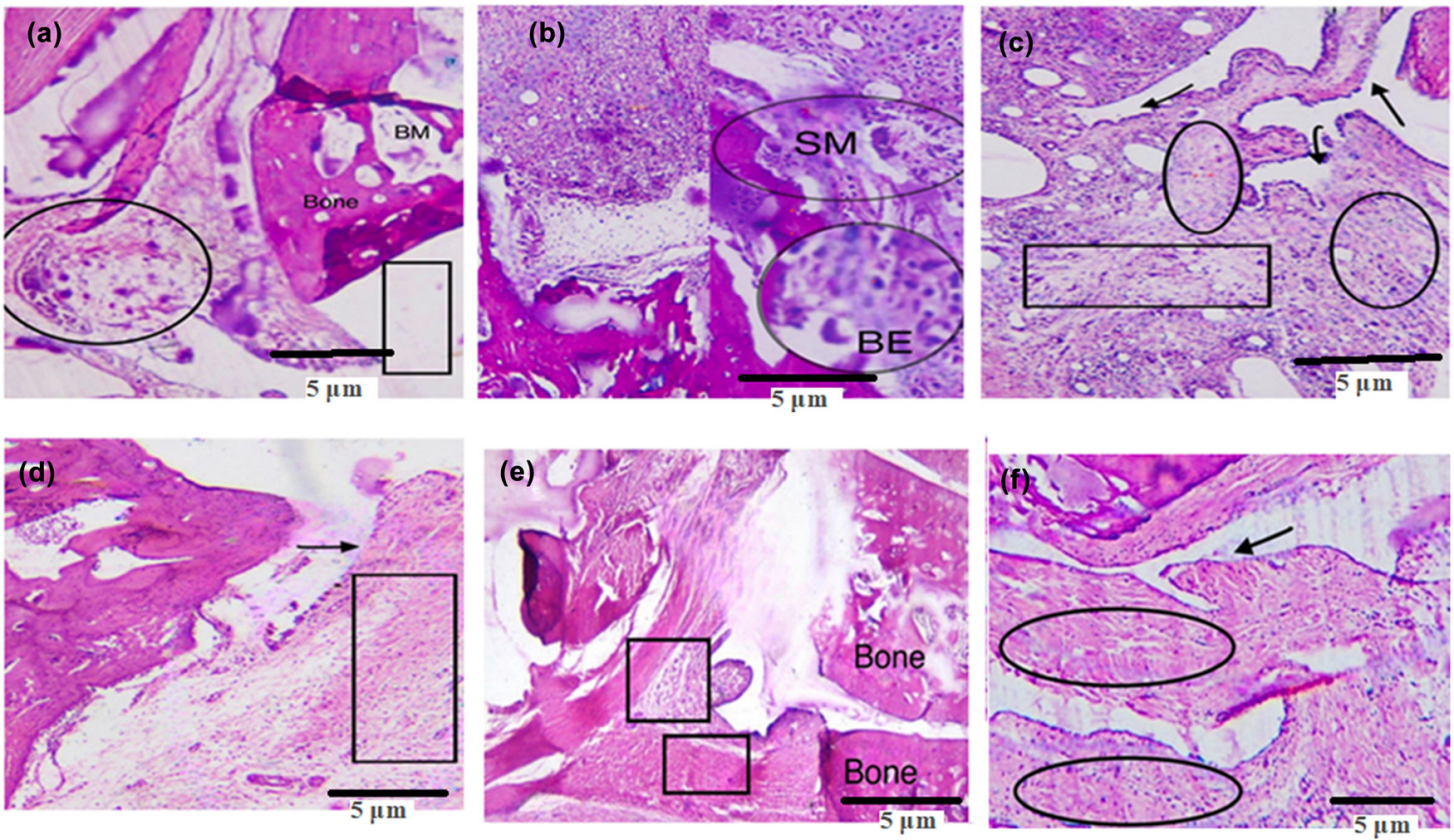
(a) Vehicle control: no pannus formation and erosion of cartilage. (b) Positive control: severe inflammation, pannus formation, and bone erosion. Inflammed synovial membrane (SM) and significant bone erosion (BE). (c) Piroxicam: moderate infiltration of inflammatory cells and pannus formation. (d) BMZ-AD 25 mg/kg: SM is surrounded by focal, dispersed inflammatory cells. (e) BMZ-AD 50 mg/kg: no inflammatory cells and pannus development. (f) BMZ-AD 75 mg/kg: no tissue changes, sloughing at the joint surface, with no infiltration of inflammatory cells and pannus formation. Scale bar is mentioned in the figure.

After treatment with BMZ-AD and piroxicam, obvious significant reduction in inflammation was observed through reduction in infiltration of inflammatory cells, pannus formation, and bone erosion (Figure 7(a)–(f)).

### 3.4 Effect of BMZ-AD on mRNA expression levels of pro-inflammatory markers

**Figure 8:**
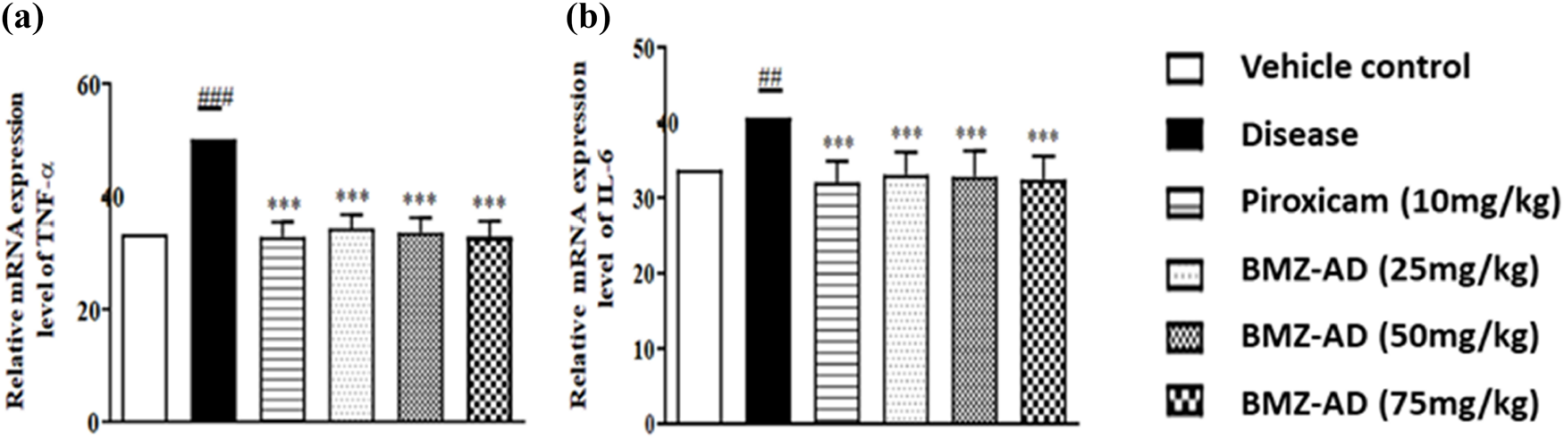
Effect of treatment with BMZ-AD on inflammatory markers (a) TNF-α and (b) IL-6. The data are expressed as mean ± SEM, while the symbol *** or ### indicates significant difference at *p* < 0.001, while ## indicates significant difference at *p* < 0.01. ### vs vehicle control, while *** vs disease group.

The disease group (50.21 ± 1.978) had substantially increased expression levels of TNF-α (*P* < 0.001) as compared to the vehicle group (33.35 ± 1.784). After treatment with piroxicam 10 mg/kg (32.86 ± 1.034), BMZ-AD 25 mg/kg (34.39 ± 0.965), BMZ-AD 50 mg/kg (33.65 ± 1.029), and BMZ-AD 75 mg/kg (32.98 ± 1.057), TNF-α levels were significantly reduced (*P* < 0.001). Nonetheless, piroxicam (32.08 ± 1.152; *P* < 0.001), BMZ-AD 25 mg/kg (33.01 ± 1.230; *P* < 0.001), BMZ-AD 50 mg/kg (32.82 ± 1.393; *P* < 0.001), and BMZ-AD 75 mg/kg (32.42 ± 1.255; *P* < 0.001) treatment also showed significant downregulation of IL-6 (Figure 8 and Table 3).

**Table 3:** BMZ-AD reduced pro-inflammatory marker mRNA expression

Biomarkers	Vehicle control	Disease	Piroxicam (10 mg/kg)	BMZ-AD (25 mg/kg)	BMZ-AD (50 mg/kg)	BMZ-AD (75 mg/kg)
Mean ± SEM						
TNF-α	33.35 ± 1.784	50.21 ± 1.978###	32.86 ± 1.034***	34.39 ± 0.965***	33.65 ± 1.029***	32.98 ± 1.057***
IL-6	33.71 ± 1.750	40.69 ± 1.189##	32.08 ± 1.152***	33.01 ± 1.230***	32.82 ± 1.393***	32.42 ± 1.255***

In comparison with the disease group BMZ-AD ***P < 0.001, while ### represents the comparison between disease and vehicle control groups.

### 3.5 BMZ-AD considerably lowered PGE2 levels

**Figure 9:**
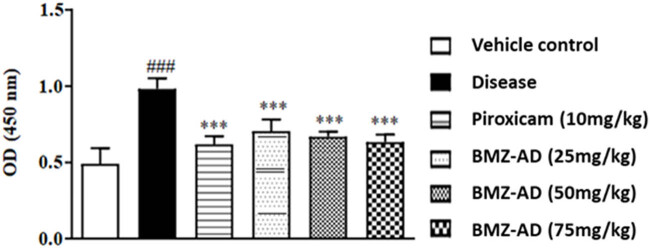
Effect of treatment with BMZ-AD on PGE2 levels. The data are expressed as mean ± SEM. The symbol *** or ### indicates significant difference at *p* < 0.001. ### vs vehicle control, while *** vs disease group.

The PGE2 value was greater (*P* < 0.001) in the disease group (0.98 ± 0.027) in contrast to the vehicle group (0.490 ± 0.040). Piroxicam (0.61 ± 0.021), BMZ-AD 25 mg/kg (0.70 ±0.030), BMZ-AD 50 mg/kg (0.665 ± 0.013), and BMZ-AD 75 mg/kg (0.631 ± 0.020) treatment groups had significantly decreased PGE2 levels, *P* < 0.001 (Figure 9).

### 3.6 Effect of BMZ-AD treatment on hematological parameters

**Figure 10:**
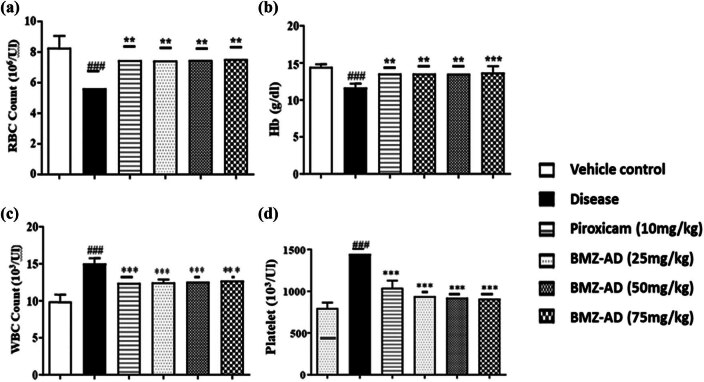
Effect of treatment with piroxicam and BMZ-AD on hematological parameters: (a) RBC count, (b) Hb content, (c) WBC count, and (d) PLT count. The data are expressed as mean ± SEM; *** and ### indicate *p* < 0.001 and ** indicates *p* < 0.01. ###*p* < 0.001 vs vehicle, while ***p < 0.001 and **p < 0.01 vs disease group.

The RBC count in the disease group (5.638 ± 0.366) was considerably decreased (*P* < 0.001) when compared to the vehicle control group (8.289 ± 0.314). RBC count was less affected (*P* < 0.01) in piroxicam (7.480 ± 0.345), BMZ-AD 25 mg/kg (7.451 ± 0.276), BMZ-AD 50 mg/kg (7.480 ± 0.273), and BMZ-AD 75 mg/kg (7.546 ± 0.256) treatment groups (Figure 10 and Table 4).

**Table 4:** BMZ-AD treatment effect on hematological parameters

Parameters	Vehicle control	Disease	Piroxicam (10 mg/kg)	BMZ-AD (25 mg/kg)	BMZ-AD (50 mg/kg)	BMZ-AD (75 mg/kg)
Mean ± SEM						
RBC (10^6^/Ul)	8.28 ± 0.314	5.63 ± 0.366###	7.480 ± 0.345**	7.451 ± 0.276**	7.480 ± 0.273**	7.546 ± 0.256**
Hb (g/dl)	14.44 ± 0.150	11.66 ± 0.213###	13.51 ± 0.291**	13.58 ± 0.337**	13.51 ± 0.336**	13.68 ± 0.359***
WBC (10^3^/Ul)	0.90 ± 0.381	15.08 ± 0.292###	12.45 ± 0.323***	12.47 ± 0.163***	12.60 ± 0.180***	12.75 ± 0.133***
PLT (10^3^/Ul)	798.83 ± 27.20	1.447 ± 15.98###	1.045 ± 35.17***	940.22 ± 17.07***	923.31 ± 17.11***	911.23 ± 19.06***

###*p* < 0.001 vs vehicle control; **p < 0.01 and ***p < 0.001 vs disease group.

Moreover, the Hb content in the disease group (11.66 ± 0.213) was considerably lower (*P* < 0.001) than in the vehicle group (14.44 ± 0.150). Piroxicam (13.51 ± 0.291; *P* < 0.01), BMZ-AD 25 mg/kg (13.58 ± 0.337; *P* < 0.01), BMZ-AD 50 mg/kg (13.5 ± 0.336; *P* < 0.01), and BMZ-AD 75 mg/kg (13.68± 0.359; *P* < 0.001) treatment groups all showed statistically remarkable increases in Hb content in contrast to the disease group (Figure 10 and Table 4).

The data showed that the WBC count decreased in treatment groups, piroxicam (12.45 ± 0.323 *P* < 0.001), BMZ-AD 25 mg/kg (12.47 ± 0.163; *P* < 0.001), BMZ-AD 50 mg/kg (12.60 ± 0.180; *P* < 0.001), and BMZ-AD 75 mg/kg (12.75 ± 0.133; *P* < 0.001), when compared to the disease group (15.08 ± 0.292) (Figure 10 and Table 4).

PLT count was practically normalized after treatment with BMZ-AD. The disease group had a higher PLT count (1,447 ± 15.98) than the vehicle control group (798.83 ± 27.20). Piroxicam (1,045 ± 35.17), BMZ-AD 25 mg/kg (940.22 ± 17.07), BMZ-AD 50 mg/kg (923.31 ± 17.11), and BMZ-AD 75 mg/kg (911.23 ± 19.06) treatment groups all demonstrated a drop in PLT count (*P* < 0.001) in contrast to the disease group (Figure 10 and Table 4).

### 3.7 Effects of BMZ-AD on biochemical parameters

#### 3.7.1 Effects of BMZ-AD on urea levels

**Figure 11:**
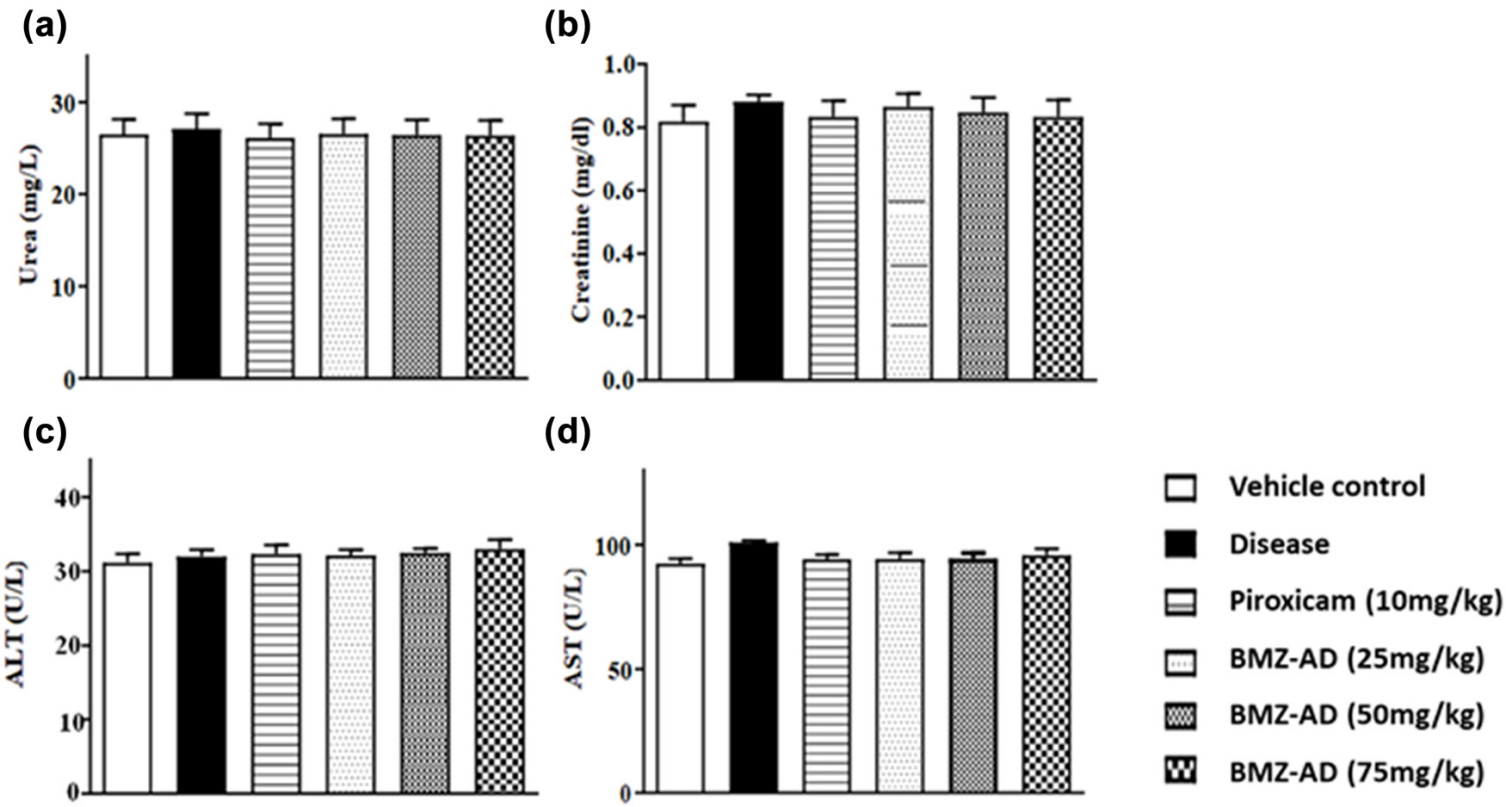
Effect of treatment with BMZ-AD on biochemical parameters: (a) urea, (b) creatinine, (c) ALT, and (d) AST levels.

In contrast to the disease group (27.11 ± 0.670), there were no variations in urea levels with piroxicam (26.14 ± 0.615), BMZ-AD 25 mg/kg (26.52 ± 0.665), BMZ-AD 50 mg/kg (26.46 ± 0.644), and BMZ-AD 75 mg/kg (26.39 ± 0.668) treatment (Figure 11 and Table 5).

**Table 5:** Biochemical parameters were not affected by BMZ-AD

Biochemical parameters	Vehicle control	Disease	Piroxicam (10 mg/kg)	BMZ-AD (25 mg/kg)	BMZ-AD (50 mg/kg)	BMZ-AD (75 mg/kg)
Mean ± SEM						
Urea (mg/dl)	26.50 ± 0.670	27.10 ± 0.67	26.12 ± 0.615	26.55 ± 0.665	26.44 ± 0.664	26.38 ± 0.668
Creatinine (mg/dl)	0.81 ± 0.213	0.88 ± 0.00	0.83 ± 0.020	0.86 ± 0.017	0.84 ± 0.018	0.83 ± 0.022
AST (IU/L)	92.50 ± 0.763	101.0 ± 0.258	94.17 ± 0.749	94.33 ± 1.02	94.50 ± 0.885	95.83 ± 1.046
ALT (IU/L)	31.17 ± 0.477	32.00 ± 0.365	32.33 ± 0.494	32.17 ± 0.307	32.50 ± 0.223	33.0 ± 0.516

#### 3.7.2 BMZ-AD effect on creatinine levels

There were no variations in creatinine levels between the disease group (0.883 ± 0.008) and the piroxicam (0.836 ± 0.020), BMZ-AD 25 mg/kg (0.866 ± 0.017), BMZ-AD 50 mg/kg (0.844 ± 0.018), and BMZ-AD 75 mg/kg (0.837 ± 0.022) treatment groups (Figure 11 and Table 5).

#### 3.7.3 Effects of BMZ-AD on ALT levels

When the vehicle control (31.19 ± 0.477), disease group (32.02 ± 0.365), and piroxicam (32.34 ± 0.494), BMZ-AD 25 mg/kg (32.19 ± 0.307), BMZ-AD 50 mg/kg (32.53 ± 0.223), and BMZ-AD 75 mg/kg (33.02 ± 0.516) treatment groups were examined; there was no significant difference in ALT levels (Figure 11 and Table 5).

#### 3.7.4 Effects of BMZ-AD on AST levels

When AST levels were assessed, there were no significant changes between the vehicle control (92.52 ± 0.763), disease group (101.03 ± 0.258), and piroxicam (94.19 ± 0.749), BMZ-AD25 mg/kg (94.35 ± 1.022), BMZ-AD 50 mg/kg (94.52 ± 0.885), and BMZ-AD 75 mg/kg (95.85 ± 1.046) treatment groups (Figure 11 and Table 5).

### 3.8 Molecular docking

**Figure 12:**
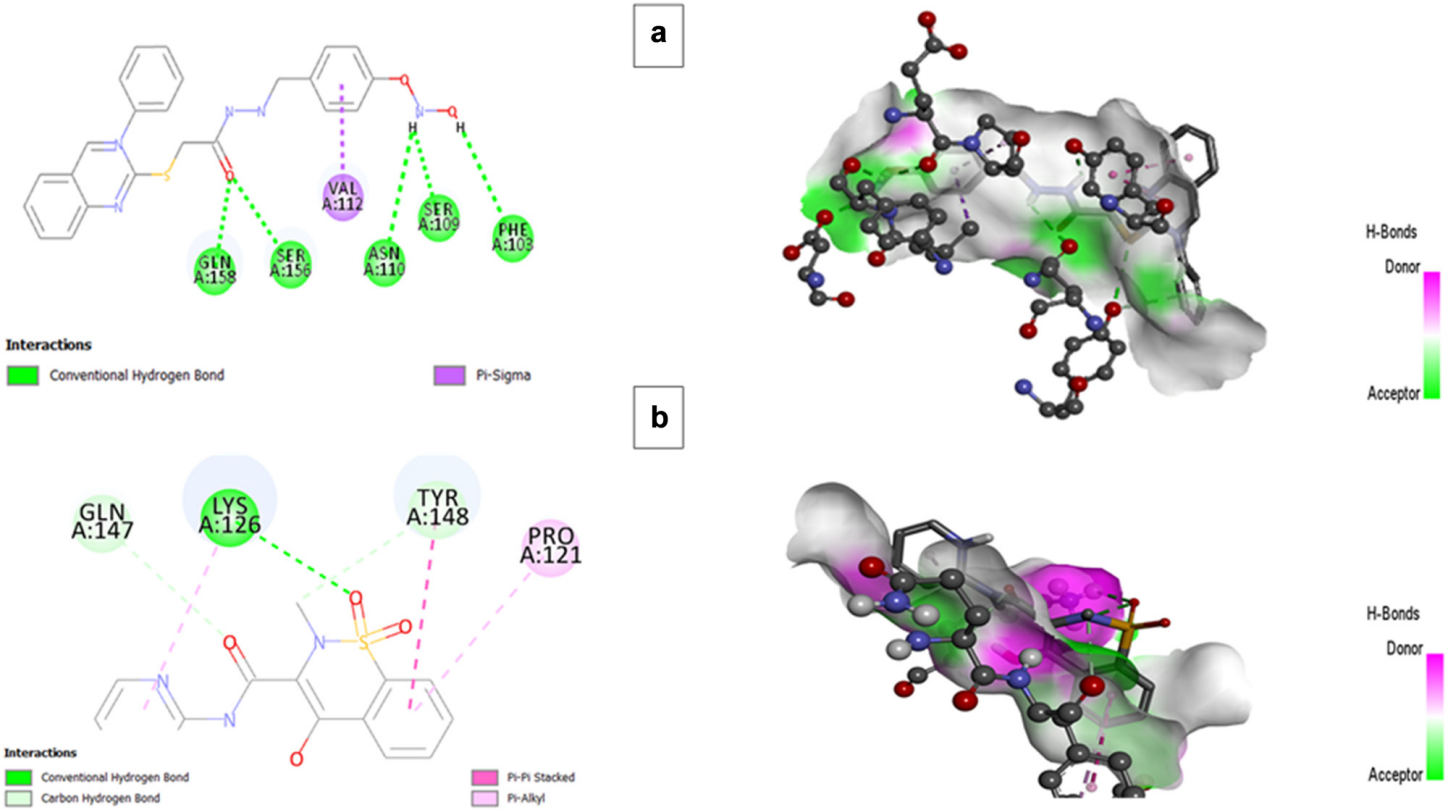
2D and 3D interactions of (a) BMZ-AD and (b) piroxicam with IL-6.

The best poses obtained from molecular docking were visualized for hydrogen bonds using Discovery Studio Visualizer. The key residues of IL-6 found to interact with BMZ-AD through hydrogen bonds included Gln158, Ser156, Asn110, Ser109, and Phe103 with a binding affinity of −6.7 (Figure 12 and Table 6).

**Table 6:** Number of hydrogen bonds with amino acid residues of IL-6 and TNF-α and binding affinity

Protein	Ligand	Binding affinity (kcal/mol)	Hydrogen bonds
IL-6	BMZ-AD	6.7	Gln158, Ser156, Asn110, Ser109, and Phe103
IL-6	Piroxicam	7.2	Lys126
TNF-α	BMZ-AD	7.1	Phe64, Glu116, Asp143, Gln61, Tyr119, and Tyr151
TNF-α	Piroxicam	8.2	Tyr151 and Leu120

**Figure 13:**
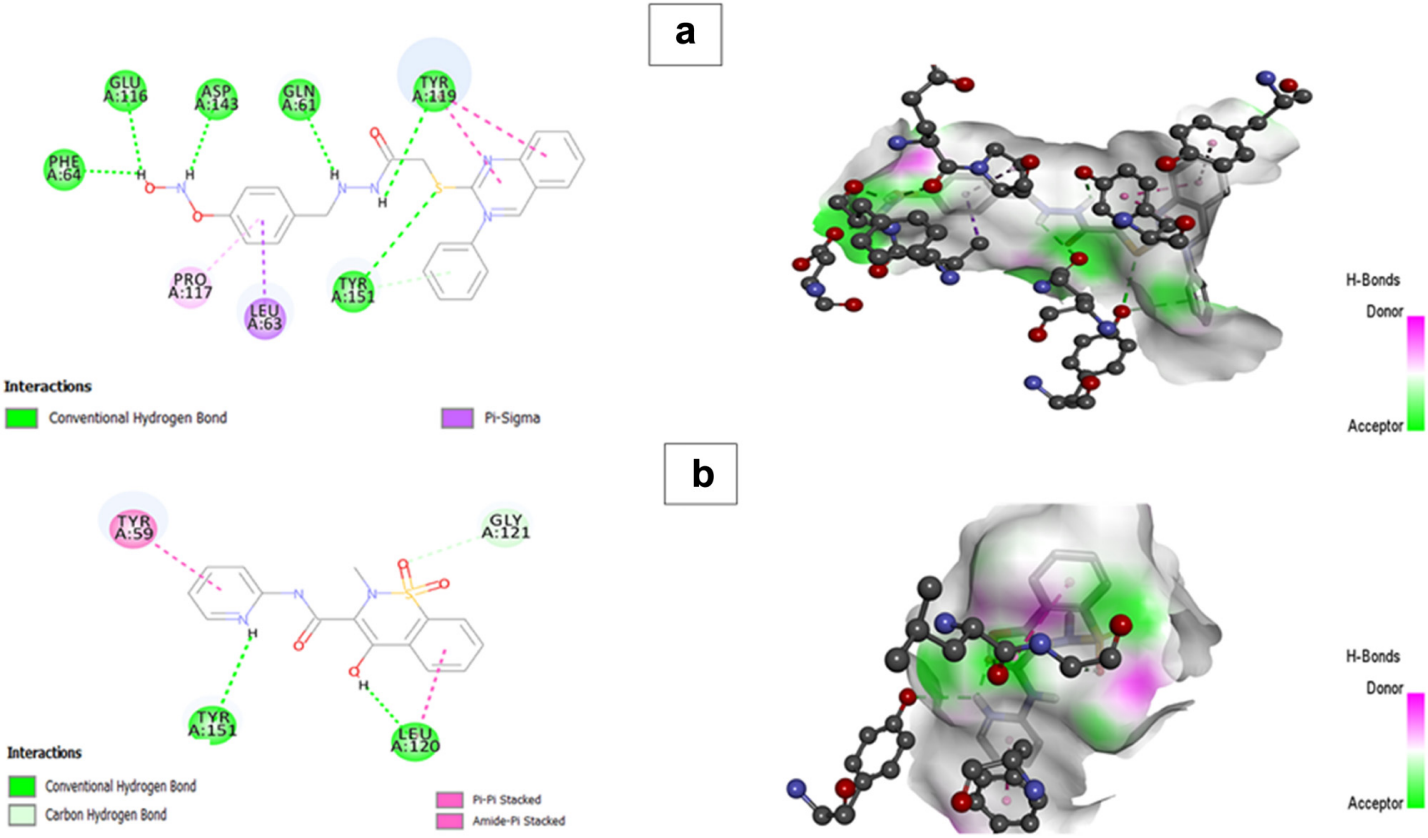
2D and 3D interactions of (a) BMZ-AD and (b) piroxicam with TNF-α.

Moreover, BMZ-AD formed hydrogen bonds with Phe64, Glu116, Asp143, Gln61, Tyr119, and Tyr151 residues of target protein TNF-α with a binding affinity of −7.1 (Table 6). When compared to the standard drug piroxicam, our drug showed better interaction and binding affinity with the target protein molecules IL-6 and TNF-α. Both bonded and non-bonded interactions between BMZ-AD and the standard drug piroxicam with the targeted proteins IL-6 and TNF-α are shown in Figures 12 and 13.

## 4 Discussion

Chronic autoimmune disease RA mainly affects the bone joints [40] and synovial lining, leading to cartilage destruction, bone erosion, disability, and increased mortality. Arthralgia, swelling, and redness are some of the clinical manifestations of RA [41]. Inflammatory cytokines are the main contributors to RA pathophysiology [42] that produce systemic effects such as acute-phase protein (CRP) formation, anemia, cardiovascular disorders, and osteoporosis [43]. The goal of current RA therapy is pain relief, inflammatory regulation, and remission of disease for all patients. However, the therapy has adverse consequences such as increased risk of heart attack and stroke after prolonged use [44], gastrointestinal disorders, dermatitis, pneumonitis, hematologic disorders, infections, nephrotoxicity, and hepatic dysregulations. Mild to severe side effects may need treatment discontinuation [44,45]. Therefore, new safer drugs are urgently required.

The FCA arthritic rat model recapitulates the immune response illicit by RA in humans. Induction of disease requires subcutaneous or intradermal administration of FCA-emulsified antigen to produce RA-like inflammation in rats [46,47]. This model mimics clinical arthritis with synovial proliferation, inflammation, and cartilage degradation [48]. In this study, we used an FCA-induced arthritic rat model to determine the anti-arthritic potential of the benzimidazole derivative BMZ-AD.

Swollen inflamed and painful joint counts are critical for RA patients to assess clinical progression of the disease and identify treatment targets [49]. Treatment with the benzimidazole derivative significantly reduced FCA-induced paw edema in rats, as compared to the disease group, after 28 days. BMD-AD 75 mg/kg produced anti-inflammatory effects comparable to our standard drug piroxicam [50]. Additionally, histopathological assessment showed a reduction in inflammatory cell infiltration after treatment with the benzimidazole derivative BMZ-AD. Pannus formation due to synovial hyperproliferation leads to cell activities that subsequently promote inflammation and disease progression [51]. BMZ-AD suppressed pannus formation significantly. The results obtained are closer to those obtained for the anti-arthritic agent piroxicam.

IL-6 and TNF-α are the primary inflammatory mediators involved in the pathogenesis of RA. High levels of IL-6 are found in the synovial fluid and serum of RA patients, which correlate with disease activity and joint degeneration [52]. As demonstrated by various studies, treatment with TNFα and IL-6 inhibitors impede the progression of joint impairment and bone erosion [53–55]. In our study, mRNA expression levels of pro-inflammatory markers IL-6 and TNFα were significantly reduced after treatment with BMZ-AD. These results were also corroborated by in silico studies where BMZ-AD bound strongly with IL-6 and TNF-α inhibitor binding sites. BMZ-AD also reduced bone erosion and inflammation aggravated by the production of PGE2-inducible enzymes [56] through suppressing the PGE2 level [57].

Chronic inflammatory processes in RA also tend to increase WBC and PLT counts because of infection and tissue damage. In this study, BMZ-AD treatment significantly reduced WBC and PLT counts, indicating its ability to affect inflammation. Additionally, BMZ-AD was helpful in increasing the RBC count and Hb concentration, indicating that it reduced the inflammatory stress that caused anemia [58]. Both Hb and RBC were found to have an inverse relationship with disease activity in RA patients [59]. In this study, BMZ-AD and the standard drug piroxicam significantly replenished the RBC count and Hb levels and reduced the WBC and PLT counts simultaneously.

Liver function impairment is prevalent in RA patients and is mostly the consequence of prolonged use of anti-rheumatic drugs. Up to 22% of the RA patients on anti-rheumatoid therapy experience abnormal LFTs, with a higher risk in alcohol consumers or having hepatic disorder [60]. AST, ALT, and ALP are reliable markers of liver as well as kidney impairment [61]. To establish the safety of BMZ-AD, we measured serum levels of ALT, AST, creatinine, and urea in all groups. The results showed no difference among all groups, suggesting that BMZ-AD does not possess hepatotoxic and nephrotoxic effects and could be marked as a safe drug.

The use of the novel benzimidazole derivative in RA treatment has its own advantages over the traditional NSAID piroxicam. Piroxicam is a non-selective COX inhibitor, and its action is only able to reduce inflammation. Literature review showed that docking results of benzimidazole derivative claimed that they can be used as COX inhibitors. The benzimidazole–NSAID conjugate showed lesser chance of side effects, especially gastrointestinal and renal complications, which are common with most NSAIDs, hence making it a safer option for patients with long-term use [62]. Moreover, benzimidazole derivatives target inflammatory cytokines, such as TNF-α and IL-6. It has been shown that such a dual effect may be able to control the disease better than piroxicam. On the basis of previous research studies which are in line with our current study, it can be concluded that BMZ-AD possesses combined anti-inflammatory and immunomodulatory properties.

Moreover, the molecular docking analysis indicates that the benzimidazole derivative BMZ-AD can form strong binding with the key proteins IL-6 and TNF-α in RA. BMZ-AD had a binding affinity of −6.7 kcal/mol toward IL-6, which involved an interaction with some important residues such as Gln158, Ser156, Asn110, Ser109, and Phe103. For TNF-α, BMZ-AD showed more pronounced binding energy of −7.1 kcal/mol, which interacted with Phe64, Glu116, Asp143, Gln61, Tyr119, and Tyr151. The results showed that BMZ-AD could target and antagonize the effect of IL-6 and TNF-α, which are vital in the RA inflammatory process. BMZ-AD had a lower affinity to bind IL-6 but had stronger interaction with TNF-α, as compared to piroxicam, which may give it a better potential for treating RA. Generally, BMZ-AD suggests it as a promising new candidate for RA treatment with significant anti-inflammatory and antiarthritic immunomodulatory potential.

## 5 Conclusions

RA presents a cascade of pathological events, such as inflammation, bone erosion, synovial hypertrophy, and loss of joint, that culminate in physical disability. The current study validates the use of a benzimidazole derivative BMZ-AD against RA using an FCA-induced arthritic model. Our data highlighted that the benzimidazole derivative possessed significant immunomodulatory and anti-inflammatory properties through attenuation of joint inflammation, bone erosion, and cartilage erosion. It might be ascribed to the downregulation of pro-inflammatory markers (TNF-α and IL-6). PGE2 levels were downregulated after treatment with the benzimidazole derivatives, while no adverse effect on renal and hepatic parameters was observed. Therefore, our findings suggest that our drug BMZ-AD can be further screened in-depth for further development in RA treatment.
